# Exercise pulmonary hypertension in patients with systemic sclerosis based on updated guidelines

**DOI:** 10.1038/s41598-024-63823-0

**Published:** 2024-06-11

**Authors:** Yu Kanazawa, Ayumi Goda, Takato Mohri, Shinsuke Takeuchi, Kaori Takeuchi, Hanako Kikuchi, Takumi Inami, Kyoko Soejima, Takashi Kohno

**Affiliations:** Department of Cardiovascular Medicine, Kyorin University Hospital, 6-20-2 Shinkawa, Mitaka, Tokyo 181-8611 Japan

**Keywords:** Cardiology, Rheumatology, Circulation

## Abstract

Recent European guidelines have introduced the concept of exercise pulmonary hypertension (ex-PH). However, the clinical characteristics of ex-PH in systemic sclerosis (SSc) remains unknown. We aimed to investigate the characteristics of exercise pulmonary hypertension (ex-PH) in patients with systemic sclerosis (SSc), which are unknown. We retrospectively examined 77 patients with SSc who underwent symptom-limited exercise testing using a cycle ergometer with right heart catheterization at our hospital. Nineteen patients with postcapillary PH were excluded. Fifty-eight patients (median age, 63 years; 55 women) were divided into the overt-PH (n = 18, mean pulmonary arterial pressure [PAP] > 20 mmHg and pulmonary vascular resistance > 2 Wood units at rest), ex-PH (n = 19, mean PAP/cardiac output slope > 3), and non-PH (n = 21) groups. Exercise tolerance and echocardiography results were compared among the groups. Peak oxygen consumption was high in the non-PH group, intermediate in the ex-PH group, and low in the overt-PH group (14.5 vs. 13.0 vs. 12.5 mL/kg/min, p = 0.043), and the minute ventilation/peak carbon dioxide production slope was also intermediate in the ex-PH group (32.2 vs. 32.4 vs. 43.0, p = 0.003). The tricuspid annular plane systolic excursion/systolic PAP ratio decreased from non-PH to ex-PH to overt-PH (0.73 vs. 0.69 vs. 0.55 mm/mmHg, p = 0.018). In patients with SSc, exercise PH may represent an intermediate condition between not having PH and overt PH, according to the new guidelines.

## Introduction

Systemic sclerosis (SSc) is a multisystem autoimmune disease characterized by vascular damage and fibrosis of the skin and internal organs. Pulmonary hypertension (PH) affects approximately 7–12% of patients with SSc and is recognized as a major cause of mortality^1–3^. The prognosis of SSc-associated PH (SSc-PH) remains poor, with a reported median survival of 4 years^4^. Specific immunosuppressive agents and selective pulmonary vasodilators have limited efficacy on SSc-PH, and therapeutic interventions that improve the prognosis have not been established^5–7^.

Recently, attention has been given to the impact of early detection of borderline PH. Borderline PH in patients with SSc is associated with worse outcomes than normal pulmonary arterial pressure (PAP)^8^. A patient with SSc with borderline PH is also highly likely to progress to overt PH^9–11^, which is recognized as "pre-pulmonary arterial pulmonary hypertension” condition^12^. Among patients with SSc with a mean PAP > 20 mmHg, one third progress to a mean PAP ≥ 25 within 3 years, with a reported mortality rate of 18% within this period^9^. The latest European Society of Cardiology (ESC)/European Respiratory Society (ERS) guidelines have introduced changes in the hemodynamic diagnostic criteria for PH^13^ by lowering the mean PAP threshold from 25 to 20 mmHg. In addition, the guidelines give a class I recommendation for annual risk assessment for PH in patients with SSc, signaling an increasing emphasis on early intervention.

Exercise testing reportedly facilitates early detection of PH, allowing for early intervention and the opportunity to improve outcomes^14–18^, as well as being useful in characterizing multifactorial exercise intolerance^19–22^. In a previous study, compared with normal exercise hemodynamics, exercise PH was associated with increased mortality among patients with SSc (5 years survival: exercise PH vs. non-PH = 82% vs. 93%)^23^. Exercise PH was also mentioned in the 2022 ESC/ERS guidelines, defined as a mean PAP/cardiac output (CO) slope > 3 mmHg/L/min^24^. Exercise PH in SSc is considered an early form of PH and is associated with the future development of PH; furthermore, it is known to be closely associated with borderline PH together with worse functional capacity^25,26^. Although exercise PH is assumed to have similar clinical significance to borderline PH, research on the characteristics of exercise PH in patients with SSc based on the new lower criteria for PH is limited. The clinical features of exercise PH in patients with SSc, such as functional capacity and right heart function, also remain unclear, and elucidating these characteristics may aid in developing a non-invasive method for early detection of SSc-PH.

Therefore, this study aimed to investigate the characteristics of exercise PH in patients with SSc who underwent exercise stress right heart catheterization (RHC), including echocardiographic parameters and exercise tolerance.

## Results

### Baseline patient characteristics

**Figure 1:**
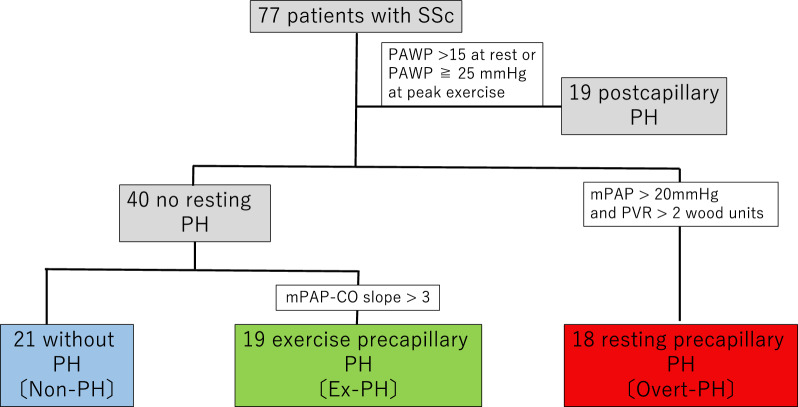
Flowchart of patients with SSc who underwent cardiopulmonary exercise testing with right heart catheterization. CO, cardiac output; ex-PH, exercise pulmonary hypertension, mPAP, mean pulmonary arterial pressure; PAWP, pulmonary arterial wedge pressure; PH, pulmonary hypertension; PVR, pulmonary vascular resistance; SSc, systemic sclerosis.

The patient flowchart is presented in Fig. 1. Among 77 eligible patients, 19 with postcapillary PH (including exercise hemodynamics), defined as pulmonary arterial wedge pressure (PAWP) > 15 mmHg at rest and/or PAWP ≥ 25 mmHg at peak exercise, were excluded from the study. Therefore, 58 patients (age, 63 [51, 71] years; men/women, 3/55) were included for analysis.

According to the definition in the 2022 ESC guidelines, 21 of the patients were included in the non-PH group, 19 in the ex-PH group, and 18 in the overt-PH group. According to a previous definition in 2015^27^, there were 47 patients without PH (mean PAP < 25 mmHg) including 15 with borderline PH (mean PAP: 21–24 mmHg). Among those 15 patients, 7 (including 4 with mean PAP/CO slope > 3 mmHg/L/min) were re-classified into the overt-PH group according to the 2022 definition.

**Figure 2:**
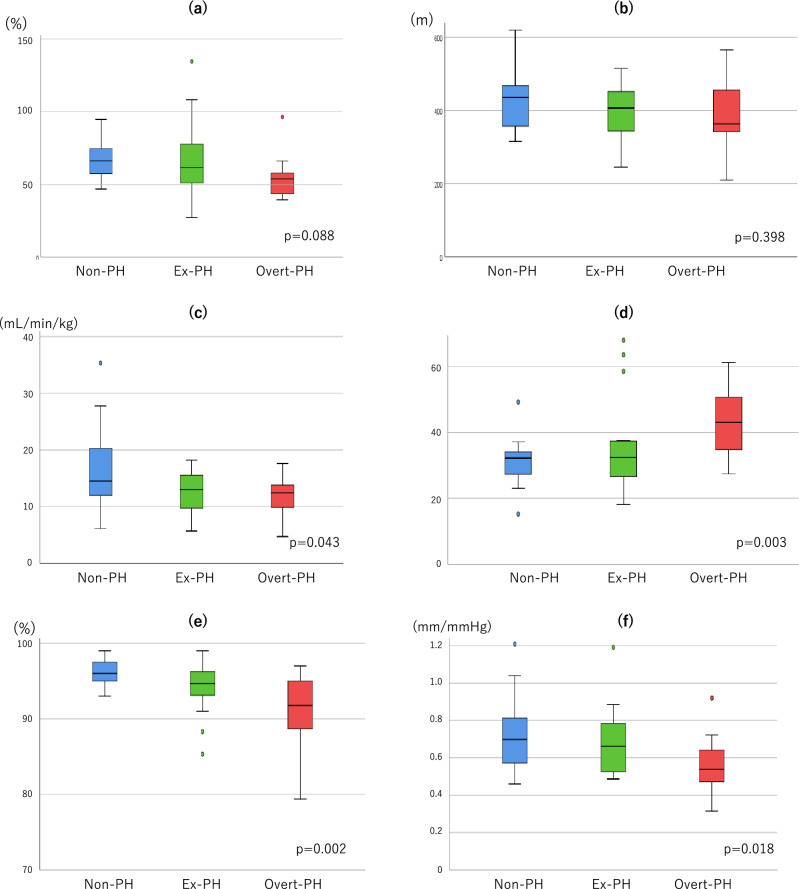
Clinical parameters across different hemodynamic subgroups (**a**) Diffusing capacity of the lung for carbon monoxide according to the alveolar volume (DLCO/VA %predicted), (**b**) 6-min walk distance, (**c**) peak oxygen consumption (VO_2_), (**d**) minute ventilation (VE) vs. peak carbon dioxide output (VCO_2_) slope, (**e**) oxygen saturation in arterial blood (SaO_2_) at peak exercise, (**f**) tricuspid annular plane systolic excursion (TAPSE)/systolic pulmonary arterial pressure (sPAP) ratio. Bars denote medians, boxes denote interquartile ranges, whiskers denote ranges excluding statistical outliers (•) (> 1.5 box lengths from either the 25th or 75th percentiles).

Baseline characteristics of the non-PH, ex-PH, and overt-PH groups, defined using the 2022 definition, are summarized in Table 1. The groups did not significantly differ in terms of age, hemoglobin and brain natriuretic peptide concentrations, comorbidities, pulmonary function (except for DLCO % predicted), or 6-min walk distance (6MWD) (Fig. 2A,B).

**Table 1:** Baseline characteristics of the included patients.

	Non-PH (n = 21)	Ex-PH (n = 19)	Overt-PH (n = 18)	p value	Non-PH vs overt-PH	Non-PH vs ex-PH	Ex-PH vs overt-PH
Age, years	56 (50–67)	65 (57–74)	65 (49–71)	0.121			
Men/Women	1/20	0/19	2/16	0.311			
Height, cm	157 (153–161)	155 (151–157)	157 (153–159)	0.529			
Body weight, kg	52 ± 14	53 ± 11	56 ± 9	0.629			
BMI, kg/m^2^	21.0 ± 4.4	21.9 ± 4.6	22.4 ± 2.7	0.536			
Six-minute walk distance, m	436 (358–466)	407 (345–452)	363 (343–447)	0.398			
Disease duration, years	8 (1–12)	9 (5–17)	15 (7–22)	0.132			
Other collagen diseases							
Sjogren syndrome, n (%)	1 (5)	3 (16)	2 (11)	0.516			
Systemic lupus erythematosus, n (%)	0 (0)	2 (11)	1 (6)	0.323			
Rheumatoid arthritis, n (%)	1 (5)	1 (5)	0 (0)	0.625			
Comorbidities							
Hypertension, n (%)	4 (19.0)	10 (52.6)	6 (33.3)	0.082			
Hyperlipidemia, n (%)	1 (4.8)	5 (26.3)	3 (16.7)	0.169			
Diabetes mellitus, n (%)	1 (4.8)	2 (10.5)	2 (11.1)	0.731			
Atrial fibrillation, n (%)	1 (4.8)	2 (10.5)	1 (5.6)	0.745			
Interstitial lung disease, n (%)	6 (28.6)	5 (26.3)	7 (38.9)	0.678			
Digestive involvement, n (%)	2 (10)	6 (32)	6 (33)	0.146			
Medications							
Immunosuppressive therapy, n (%)	11 (52)	9 (50)	13 (72)	0.327			
ERA, n (%)	3 (14)	2 (11)	11 (61)	0.001			
PDE5i, n (%)	2 (10)	2 (11)	3 (17)	0.767			
SGCS, n (%)	0 (0)	0 (0)	8 (44)	< 0.001			
Oral prostacyclin, n (%)	1 (5)	0 (0)	6 (33)	0.003			
Intravenous prostacyclin, n (%)	0 (0)	2 (11)	0 (0)	0.119			
Blood analysis							
BNP, pg/dL	19 (12–40)	37 (17–63)	26 (15–45)	0.374			
Hemoglobin, g/dL	12.5 ± 1.3	12.5 ± 1.0	12.6 ± 1.5	0.988			
Autoantibodies (n = 55)							
Anti-nuclear + , n (%)	16 (76)	16 (89)	13 (81)	0.590			
Anti-centromere + , n (%)	6 (29)	10 (56)	6 (38)	0.223			
Anti-SCL70 + , n (%)	5 (24)	0 (0)	0 (0)	0.012			
Pulmonary function (n = 52)							
FEV1% predicted, %	84 (76–94)	86 (70–100)	78 (62–83)	0.809			
FVC % predicted, %	88 (75–92)	93 (74–103)	70 (63–92)	0.150			
DLCO % predicted, %	61 (50–67)	61 (51–65)	42 (38–48)	0.003	0.011	1.000	0.010
DLCO/VA % predicted, %	66 (58–75)	62 (51–79)	54 (44–58)	0.088			
%FVC/%DLCO	1.5 (1.2–1.9)	1.6 (1.3–1.9)	2.0 (1.3–2.2)	0.399			
Hemodynamic parameters (flat position)							
Mean RAP, mmHg	3 (2–4)	2 (1–3)	5 (4–5)	0.002	0.077	0.094	0.006
Systolic PAP, mmHg	28 ± 5	29 ± 7	41 ± 5	< 0.001	< 0.001	1.000	< 0.001
Diastolic PAP, mmHg	6 ± 4	6 ± 4	12 ± 4	< 0.001	< 0.001	1.000	< 0.001
Mean PAP, mmHg	15 ± 3	17 ± 4	25 ± 3	< 0.001	< 0.001	0.138	< 0.001
PAWP, mmHg	6 (4–7)	6 (4–7)	7 (5–9)	0.207			
SaO_2_, %	97 (96–98)	95 (95–97)	95 (93–96)	0.001	0.002	0.038	0.882
SvO_2_, %	72 ± 5	72 ± 6	72 ± 5	0.996			
Cardiac output, L/min	4.0 (3.4–5.1)	4.3 (3.8–5.2)	4.9 (4.1–5.2)	0.177			
PVR, Wood units	1.9 (1.5–2.5)	1.9 (1.7–3.2)	3.5 (3.1–4.0)	< 0.001	0.001	1.000	0.011

Values are reported as mean ± standard deviation or medians (interquartile ranges) or n (%).

*BMI* body mass index, *BNP* brain natriuretic peptide, DLCO/VA % predicted, diffusing capacity of the lung for carbon monoxide, normalized according to alveolar volume, *ERA* endothelin receptor antagonist, *ex-PH *exercise pulmonary hypertension, *PAP* pulmonary arterial pressure, *PAWP* pulmonary arterial wedge pressure, *PDE5i* phosphodiesterase type V inhibitor, *PH* pulmonary hypertension, *PVR* pulmonary vascular resistance, *RAP* right atrial pressure, *SaO*_*2*_ O_2_ saturation in arterial blood, *SGCSs* soluble guanylate cyclase stimulator, *SvO*_*2*_ O_2_ saturation in the pulmonary artery, *%FEV1* forced expiratory volume in 1 s %predicted, *%FVC* forced vital capacity %predicted, *%DLCO* diffusing capacity of the lung for carbon monoxide %predicted.

Patients in the overt- and ex-PH groups had a lower resting oxygen (O_2_) saturation in the arterial blood (SaO_2_) than those in the non-PH group (non-PH vs. ex-PH vs. overt-PH = 97% [96%, 98%] vs. 95% [95%, 97%] vs. 95% [93%, 96%], p = 0.001).

### Exercise hemodynamic parameters

The results of cardiopulmonary exercise testing (CPX) with RHC at rest (after leg raise) and peak in each group are summarized in Table 2. Among the CPX parameters, peak O_2_ consumption (peak VO_2_; non-PH vs. ex-PH vs. overt-PH = 14.5 [12.0, 20.3] vs. 13.0 [9.8, 15.6] vs. 12.5 [10.1, 13.7] mL/kg/min, p = 0.043) and minute ventilation (VE) vs. carbon dioxide output (VCO_2_) slope (non-PH vs. ex-PH vs. overt-PH = 32.2 [27.3, 34.1] vs. 32.4 [26.7, 37.4] vs. 43.0 [34.8, 50.7], p = 0.003) were significantly different between the three groups (Fig. 2C,D). SaO_2_ at peak exercise was also significantly different between the three groups (non-PH vs. ex-PH vs. overt-PH = 96% [95%, 98%] vs. 95% [93%, 96%] vs. 92% [89%, 95%], p = 0.002) (Fig. 2E). Among exercise parameters, peak VO_2_, VE vs. VCO_2_ slope, and SaO_2_ at peak exercise were intermediate during ex-PH, between those in non-PH and overt-PH.

**Table 2:** Hemodynamic parameters at rest (after leg raise) and peak exercise.

	Non-PH (n = 21)	Ex-PH (n = 19)	Overt-PH (n = 18)	p value	Non-PH vs Overt-PH	Non-PH vs Ex-PH	Ex-PH vs Overt-PH
Rest after leg raise							
HR, bpm	72 ± 14	71 ± 14	76 ± 10	0.523			
Systolic PAP, mmHg	29 ± 6	35 ± 7	48 ± 8	< 0.001	< 0.001	0.036	< 0.001
Diastolic PAP, mmHg	8 ± 3	8 ± 4	14 ± 4	< 0.001	< 0.001	1.000	< 0.001
Mean PAP, mmHg	18 ± 4	21 ± 6	29 ± 4	< 0.001	< 0.001	0.058	< 0.001
PAWP, mmHg	8 (7–13)	9 (7–13)	10 (7–13)	0.993			
Cardiac output, L/min	4.6 (3.8–6.0)	5.2 (4.0–6.2)	6.4 (5.1–8.9)	0.012	0.016	1.000	0.079
PVR, Wood units	2.0 ± 1.4	2.4 ± 1.1	3.1 ± 1.1	0.025	0.021	0.890	0.275
PAC, mL/mmHg	3.1 (2.0–5.2)	2.5 (2.2–3.7)	2.7 (2.4–3.0)	0.593			
C(a-v)O_2_, mL/dL	4.4 ± 0.7	4.3 ± 1.1	3.7 ± 0.7	0.027	0.029	1.000	0.137
SaO_2,_ %	98 (96–98)	97 (96–98)	95 (92–97)	0.002	0.002	0.675	0.096
SvO_2,_ %	70 ± 5	70 ± 6	72 ± 5	0.641			
VO_2_, mL/min	204 ± 68	216 ± 51	250 ± 47	0.046	0.048	1.000	0.226
VCO_2_, mL/min	175 ± 59	179 ± 40	218 ± 52	0.022	0.033	1.000	0.072
R	0.85 ± 0.06	0.83 ± 0.08	0.83 ± 0.09	0.867			
VE, L/min	7.8 ± 2.3	7.7 ± 1.5	10.4 ± 2.3	< 0.001	0.001	1.000	0.001
VE/VO_2_	38.6 (34.9–40.9)	38.1 (30.9–40.7)	42.3 (34.9–50.2)	0.163			
VE/VCO_2_	46.9 (39.8–51.7)	42.3 (39.2–49.7)	50.1 (42.4–61.5)	0.179			
Peak							
Work rate, W	64 ± 21	48 ± 20	48 ± 22	0.027	0.065	0.061	1.000
HR, bpm	114 ± 26	109 ± 19	108 ± 21	0.709			
Systolic PAP, mmHg	49 ± 10	60 ± 11	70 ± 12	< 0.001	< 0.001	0.005	0.018
Diastolic PAP, mmHg	13 ± 4	18 ± 7	19 ± 8	0.014	0.020	0.062	1.000
Mean PAP, mmHg	29 ± 6	38 ± 7	44 ± 8	< 0.001	< 0.001	< 0.001	0.034
PAWP, mmHg	16 (10–21)	18 (16–22)	16 (10–18)	0.261			
Cardiac output, L/min	9.9 (9.1–12.6)	8.3 (6.7–11.0)	9.6 (8.2–11.6)	0.303			
PVR, Wood units	1.1 (0.8–1.6)	2.3 (2.0–3.1)	3.0 (2.1–4.2)	< 0.001	< 0.001	< 0.001	0.498
PAC, mL/mmHg	2.7 (2.1–3.4)	2.1 (1.7–2.4)	1.9 (1.6–2.1)	0.004	0.006	0.041	1.000
C (a-v) O_2_, mL/dL	8.2 ± 1.5	7.6 ± 1.7	6.7 ± 1.3	0.017	0.014	0.743	0.253
SaO_2_, %	96 (95–98)	95 (93–96)	92 (89–95)	0.002	0.002	0.146	0.146
SvO_2_, %	46 ± 8	49 ± 9	51 ± 7	0.199			
VO_2_, mL/min	850 (656–1096)	673 (509–772)	685 (603–794)	0.057			
VCO_2_, mL/min	924 (635–1240)	684 (540–880)	756 (613–810)	0.164			
R	1.07 ± 0.18	1.10 ± 0.13	1.06 ± 0.13	0.722			
VE, L/min	27.8 (24.5–39.3)	27.6 (21.4–30.4)	33.2 (29.6–39.3)	0.053			
VE/VO_2_	38.5 (32.4–42.2)	41.9 (32.4–49.1)	50.9 (44.4–58.2)	0.009	0.004	0.873	0.242
VE/VCO_2_	37.0 ± 8.2	40.0 ± 11.0	48.6 ± 9.3	0.001	0.001	0.981	0.024
Peak VO_2,_ mL/kg/min	14.5 (12.0–20.3)	13.0 (9.8–15.6)	12.5 (10.1–13.7)	0.043	0.123	0.082	1.000
VE vs. VCO_2_ slope	32.2 (27.3–34.1)	32.4 (26.7–37.4)	43.0 (34.8–50.7)	0.003	0.002	1.000	0.044

Values are reported as mean ± standard deviation or medians (interquartile ranges).

*C (a-v) O*_*2*_ arteriovenous O_2_ difference, *ex-PH* exercise pulmonary hypertension, *HR* heart rate, *PAC* pulmonary arterial compliance, *PAP* pulmonary arterial pressure, *PAWP* pulmonary arterial wedge pressure, *PH* pulmonary hypertension, *PVR* pulmonary vascular resistance, *SaO*_*2*_ O_2_ saturation in arterial blood, *SvO*_*2*_ O_2_ saturation in the pulmonary artery, *VO*_*2*_ oxygen consumption, *VCO*_*2*_ carbon dioxide output, *R* respiratory exchange ratio, *VE* minute ventilation.

### Echocardiographic parameters

Echocardiographic parameters in the groups are summarized in Table 3. Among the echocardiographic parameters, the TASPE/systolic PAP (sPAP) ratio was significantly different among the three groups (non-PH vs. ex-PH vs. overt-PH = 0.73 ± 0.20 vs. 0.69 ± 0.19 vs. 0.55 ± 0.15 mm/mmHg, p = 0.018) (Fig. 2F). The other parameters representing right ventricular (RV) function were not significantly different among the groups.

**Table 3:** Echocardiographic parameters.

	Non-PH (n = 21)	Ex-PH (n = 19)	Overt-PH (n = 18)	p value	Non-PH vs Overt-PH	Non-PH vs Ex-PH	Ex-PH vs Overt-PH
LVEF, %	65 ± 4	66 ± 5	62 ± 9	0.132			
LAD, mm	32 (30–36)	33 (27–38)	31 (27–36)	0.972			
LAVi, mL/m^2^	31 (26–36)	34 (28–38)	32 (27–46)	0.859			
E, cm/s	67.7 ± 11.1	62.1 ± 15.0	64.8 ± 18.3	0.521			
A, cm/s	69.7 ± 16.9	78.9 ± 14.6	76.1 ± 17.8	0.233			
E/A	1.0 (0.8–1.2)	0.8 (0.7–1.0)	0.8 (0.7–0.9)	0.059			
DcT, cm/s	182 ± 45	218 ± 54	228 ± 37	0.010	0.013	0.057	1.000
e’ septal, cm/s	7.4 ± 2.1	6.2 ± 1.7	6.0 ± 2.1	0.090			
E/e’ septal, cm/s	9.8 ± 2.8	10.5 ± 3.1	11.3 ± 3.7	0.386			
TVs’, cm/s	11.7 ± 1.7	12.8 ± 2.0	12.3 ± 2.1	0.244			
TAPSE, mm	22 ± 3	22 ± 3	22 ± 4	0.913			
RV FAC, %	41 ± 7	43 ± 7	38 ± 4	0.110			
TAPSE/sPAP, mm/mmHg	0.73 ± 0.20	0.69 ± 0.19	0.55 ± 0.15	0.018	0.019	0.857	0.095

Values are reported as mean ± standard deviation or medians (interquartile ranges).

*A* late peak diastolic velocity of the mitral inflow, *DcT* deceleration time, *E* early peak diastolic velocity of the mitral inflow, e’ septal mitral annular motion with the sample volume placed in the septal, *ex-PH* exercise pulmonary hypertension, *LAD* left atrial diameter, *LAVi* left atrium volume index, *LVEF* left ventricular ejection fraction, *PH* pulmonary hypertension, *RV FAC* right ventricular fractional area change, *sPAP* systolic pulmonary arterial pressure, *TAPSE* tricuspid annular plane systolic excursion, *TVs*’ peak systolic velocity of tricuspid annulus.

## Discussion

We have elucidated the impact and characteristics of the exercise PH in the context of the new definition. Most patients who would be diagnosed with borderline PH according to the old definition had ex-PH and were re-classified as overt-PH under the new definition. Based on the results of the 6-min walk test (6MWT) and CPX, exercise tolerance in the ex-PH group was intermediate, between those of the non-PH and overt-PH groups. In addition, the tricuspid annular plane systolic excursion (TAPSE)/sPAP ratio upon echocardiography, which is an RV-pulmonary arterial coupling index, was also intermediate in the ex-PH group. Exercise testing with RHC is necessary for the diagnosis of ex-PH, and assessments with other non-invasive tests remain to be developed. Monitoring and assessment of these parameters may contribute to the early detection and management of PH in patients with SSc, which could improve their clinical outcomes and quality of life. Further research is warranted to validate these results and explore their clinical implications in larger patient cohorts.

### Exercise tolerance

In the context of SSc, CPX is a valuable non-invasive tool for the detection of PH. In a previous report, a peak VO_2_ of 18.7 mL/kg/min or higher was effective to rule out SSc-PH, with a high negative predictive value^28^. Similarly, in our study, exercise tolerance indicators, such as 6MWD and peak VO_2_, were gradually decreased from a non-PH state to ex-PH and overt-PH.

However, exercise tolerance in patients with SSc is influenced by several factors and does not reflect hemodynamics alone^19^. In our current study, even patients with SSc without PH had a 6MWD of 436 m and peak VO_2_ of 14.5 mL/kg/min, corresponding to the “intermediate risk” category in the comprehensive risk assessment^13^. Importantly, exercise tolerance is generally lower in patients with SSc, who are predominantly older, than in those with other forms of PH. It is well recognized that SSc, as a systemic disease, leads to reduced exercise capacity due to peripheral/muscular limitations and impaired tissue oxygen extraction^19,20^. Unlike peak VO_2_, which is a measure of maximal load, the 6MWD is a measure of submaximal load and is affected by multifactorial exercise intolerance, such as peripheral effects, which may be one reason for the lack of statistically significant differences in 6MWD among the groups in our study.

The elevated VE vs. VCO_2_ slope in our study indicates abnormal ventilation/perfusion matching and increased dead space ventilation, both of which are associated with pulmonary vascular dysfunction and impaired gas exchange^29^. Previous reports also suggest that indicators of ventilatory inefficiency such as VE vs. VCO_2_ slope are useful for detecting PH in SSc, and our results similarly positioned ex-PH between non-PH and overt-PH^28^. Unlike peak VO_2_, the VE vs. VCO_2_ slope in ex-PH corresponds to the “low risk” category in the PH risk classification guidelines^13^. Because the VE vs. VCO_2_ slope can be evaluated even at submaximal effort, it may provide a more accurate assessment of risk category than 6MWD and peak VO_2_, which are sensitive to deconditioning in SSc.

### TAPSE/sPAP ratio

The invasive nature of exercise stress testing with RHC poses a challenge owing to the limited availability of facilities capable of performing the test. In contrast, echocardiography is a non-invasive and effective screening tool.

In the recent ESC/ERS guidelines for PH, the established cut-off values for PH screening and key echocardiographic parameters did not change substantially. However, a novel indicator of PH, the TAPSE/sPAP ratio, was introduced^13^. This ratio combines measures of RV function and pulmonary artery indices, easily assessable via standard echocardiography, serving as an estimate of RV-pulmonary artery coupling^30^. In PH, progressive pulmonary vascular remodeling augments the load on the contracting RV, consequently modifying RV-pulmonary arterial coupling. A cut-off value of the TAPSE/sPAP ratio for PH screening has been proposed based on a cohort study^31^, leading to its inclusion as a screening indicator in the updated guidelines. More recent studies have demonstrated its utility in risk stratification and prognostication of patients with PH, including those with SSc^32,33^. In the SSc EUSTAR cohort, a TAPSE/sPAP ratio < 0.55 mm/mmHg was identified as a risk factor for PH^34^.

In our study, the TAPSE/sPAP ratio effectively differentiated ex-PH from non-PH and overt-PH. This suggests its potential as an early detection marker for SSc-PH. In our cohort, the TAPSE/sPAP ratio was 0.66 mm/mmHg for ex-PH compared to 0.54 mm/mmHg for overt-PH. Furthermore, in SSc, the TAPSE/sPAP ratio reportedly correlates with peak VO_2_ and the VE vs. VCO_2_ slope, parameters derived from CPX, supporting our results that CPX parameters and the TAPSE/sPAP ratio are associated with hemodynamic severity^35^.

This study had several limitations. It was a retrospective, single-center study, including a relatively small sample size; therefore, the statistical power may not have been sufficient to detect negative outcomes. The study population did not include patients with overt or occult left ventricular diastolic dysfunction. Additionally, some patients with SSc were unable to undergo exercise stress tests owing to orthopedic issues, sarcopenia, and other factors, leading to their exclusion from the study. Our cohort was exclusively Japanese and comprised older and predominantly female patients; therefore, the results of this study may not be generalizable to younger adults or men. Age is known as an important confounder of the pulmonary vascular response during exercise and exercise tolerance, such as peak VO_2_ and 6MWD.

The definition of exercise PH has been established, and its association with poor prognosis in the population, including patients with SSc, is well-known^23,36,37^. However, the prognostic impact of the updated definition, where borderline PH (mean PAP: 21–24 mmHg) is now classified as overt PH, remains unclear. Assessing its prognostic impact requires long-term observation and further research exploration.

In conclusion, in patients with SSc, exercise PH may represent an intermediate condition between not having PH and overt PH, according to the new guideline definitions.

## Methods

### Study participants

In this retrospective, cross-sectional study, we examined consecutive patients with SSc who exhibited dyspnea during exertion, performed owing to suspected or confirmed PH, and who underwent CPX with RHC at our hospital from 2013 to 2022. Definitions of SSc were based on the American College of Rheumatology diagnostic criteria^38^.

This study was approved by the Committee for Clinical Studies and Ethics of Kyorin University School of Medicine. The purposes and risks of the study were explained to the patients, who provided informed consent prior to participating. All methods were performed in accordance with the relevant guidelines and regulations.

### RHC and CPX

RHC was performed with a 6-F, double-lumen, balloon-tipped, flow-directed Swan–Ganz catheter (Harmac Medical Products, Inc., Buffalo, NY, USA) via a transjugular approach, as previously described^39^. Baseline hemodynamic data were recorded, and the zero-reference level (mid-chest) was adjusted at the start of pressure measurement. Measurements were obtained at the end of normal expirations with patients in the supine position to assess the right ventricular pressure, mean PAP, sPAP, diastolic PAP, and PAWP.

Incremental, symptom-limited CPX was performed with patients in the supine position by using an electromagnetically braked cycle ergometer (Nuclear Imaging Table with Angio Ergometer; Lode BV, Groningen, The Netherlands) and the ramp protocol. The test comprised a 3-min rest period, followed by 3-min warm-up at an ergometer setting of 10 W (60 rpm), and testing with 1-W increase in exercise load every 6 s (totaling 10 W/min). During the exercise, VO_2_, VCO_2_, and VE were measured using a metabolic cart (Cpex-1; Inter Reha Co. Ltd., Tokyo, Japan). Peak VO_2_ was calculated as the average value obtained during the last 30 s of exercise. The VE vs. VCO_2_ slope was calculated from the start of incremental exercise to the respiratory compensation point by using least-squares linear regression.

Heart rate, arterial blood pressure directly recorded in the radial artery, and electrocardiogram were monitored continuously during the test. PAP and PAWP in the RHC were measured every minute. We used the averaged mean PAP and mean PAWP during several-second periods rather than end-expiratory measurements during exercise. SaO_2_ and the pulmonary artery (SvO_2_) were measured at rest and during warm-up, submaximal, and peak exercise. CO was determined via the Fick method, using the following formula: CO (L/min) = VO_2_/[1.34 × hemoglobin concentration (Hb) × (SaO_2_ − SvO_2_)]. The arterial mixed venous oxygen content difference was calculated as follows: 13.4 × Hb × (SaO_2_ − SvO_2_)/1000. Pulmonary vascular resistance (PVR) and pulmonary arterial compliance (PAC) were calculated as: PVR (Wood units) = (mean PAP – PAWP)/CO and PAC = stroke volume/pulse pressure. The mean PAP/CO slope was calculated from multipoint plots of the mean PAP and CO by using least-squares linear regression^40^.

The 6MWT was performed according to American Thoracic Society guidelines without supplemental O_2_ one day before RHC^41^.

### Echocardiography

A transthoracic Doppler echocardiogram was obtained with the patient in the resting state and digitally stored on an Artida (Toshiba, Tokyo, Japan) or EPIQ (Philips Healthcare, Cambridge, MA USA) ultrasound system within 3 months of the RHC. Left ventricular ejection fraction was calculated using Simpson’s biplane method in the apical four- and two-chamber views. Left atrial volume was measured in the same way and was indexed according to body surface area. Mitral inflow was assessed in the apical four-chamber view with the pulsed-wave Doppler sample volume placed at the tips of the mitral valve leaflets during diastole; accordingly, the early (E) and late peak diastolic velocities of the mitral inflow and the E-wave deceleration time were measured. Mitral annular motion was assessed using pulsed-wave tissue Doppler imaging with the sample volume placed in the septal (e’ septal). Subsequently, the E/e’ ratio was calculated. Tricuspid regurgitation peak velocity and inferior vena cava diameter (right atrial pressure estimation) were used to calculate sPAP by using the Bernoulli equation in the absence of laminar tricuspid regurgitation and/or pulmonary stenosis. The RV systolic function was assessed by measuring TAPSE. Subsequently, the TAPSE/sPAP ratio was calculated. The RV end-diastolic area (RVEDarea) and end-systolic area (RVESarea) were assessed via manual planimetry in the apical four-chamber view, and the RV fractional area change (RVFAC) was derived using the formula: RVFAC = [(RVEDarea-RVESarea)/RVEDarea] × 100.

### Hemodynamic definitions

Based on the 2022 ESC guidelines^13^, patients with SSc were divided into three different groups according to their resting and exercise hemodynamics: 1) overt PH (mean PAP at rest > 20 mmHg and PVR at rest > 2 Wood units; 2) exercise PH (ex-PH, mean PAP/CO slope > 3 mmHg/L/min); and 3) non-PH (without PH) groups.

### Statistical analysis

Continuous variables are presented as mean ± standard deviation or medians (25^th^ quartile, 75^th^ quartile). The Shapiro–Wilk test and histogram analyses were performed to assess datasets for normality. Comparisons of more than two groups were performed using a one-way analysis of variance (with the Tukey post-hoc test) or the Kruskal–Wallis test (with Dunn’s post-hoc test), as appropriate. Categorical variables are presented as frequencies (percentages) and were compared using the Fisher’s exact test or Pearson’s χ^2^ test. Analyses were performed using SPSS Statistics for Windows (version 26.0; IBM Corp., Armonk, NY, USA).

## Data Availability

All data generated or analyzed during this study are included in this article. Further inquiries can be directed to the corresponding authors.
